# Finite-Element-Analysis-Based Study of a Failure Phenomenon in HDPE Pipes

**DOI:** 10.3390/ma16216944

**Published:** 2023-10-29

**Authors:** Horatiu Teodorescu Draghicescu, Maria Luminita Scutaru, Sorin Vlase

**Affiliations:** 1 Department of Mechanical Engineering, Transylvania University of Brasov, B-Dul Eroilor, 29, 500036 Brasov, Romania; hteodorescu@unitbv.ro (H.T.D.); svlase@unitbv.ro (S.V.); 2 Romanian Academy of Technical Sciences, Bulevardul Dacia 26, București, 030167 Bucharest, Romania

**Keywords:** parrot’s beak failure, HDPE, pipes, water supply network, FEM

## Abstract

In pipes made of HDPE used in city water supply networks, a specific type of failure is commonly noted, called the parrot’s beak failure. It requires expensive intervention. The prediction and study of the development of this defect, therefore, requires thorough research. In this work, the finite element method is used to study the mechanism of the occurrence and development of this defect. Two examples of the calculation for the concrete case of some tubes used in a water supply network are presented. This study is important for the designers of such networks, to predict and prevent the occurrence of this defect that can lead to unwanted network downtime and high repair costs.

## 1. Introduction

Water supply networks exist in all human settlements, and the tubes used in these supply networks represent a very important element in the creation of these networks. Drinking water distribution systems play a particularly important role. These are made up of pipe networks, storage basins, pumps, and other accessories necessary for the proper functioning of the system. There must also be a command and control system that must meet multiple requirements.

Such a system is served by numerous pipes, with very large diameters at the entrance to the system, starting from the water purification stations and continuing with pipes of increasingly smaller diameters, which go to the final consumers. Towards the point of public use, these pipes have a diameter of approximately 150 mm, thus allowing for the connection with the final consumers (public buildings, houses, industrial consumers, etc.). Worldwide, the total length of these pipes is enormous, so the importance of their design and manufacture is huge. Thus, the efforts made by researchers to study the behavior of pipes during water transport is justified. These pipes must meet many conditions, withstand high loads, and have a long service life, as repairs are very expensive.

Pipes for transporting water have been made, throughout history, from different materials, corresponding to the level of technological development of the society. At the moment, pipes made of asbestos cement, cast iron, ductile iron, plastic, reinforced concrete, and steel are in service.

The creation of polyethylene (PE) has offered multiple advantages to this field: high-volume and low-price production; simple modeling; the possibility of ensuring a simple and integrated design (multifunctional components, such as couplings and fittings); simple transport, handling, and assembly; easy maintenance; good mechanical resistance and resistance to seismic events; chemical and corrosion resistance; a very good lifespan; very good operation at low temperatures; and the possibility of recycling the materials after use. At the moment, pipes made of high-density polyethylene (HDPE) represent the ideal solution for human water supply systems. The first applications date back to the 1960s. HDPE proved to be a hard, very resistant material with long durability, very good handling, and an excellent price (the lowest lifetime cost among the previously mentioned materials [1]).

HDPE is currently the standard material used in water transport, and represents a modern and economical solution, as well as being the most suitable plastic [1,2]. Tubes and fittings manufactured from this material have the appropriate mechanical properties, a long life, and low manufacturing and maintenance costs. The manufacture of these materials is in its third generation [3]. Pipes made of these materials have good mechanical properties, being able to withstand variable loads, as currently happens in the case of water supply networks. Their fracture, in the case of the ductile fracture mode, is in the form of a parrot’s beak [4,5].

The mechanical behavior of HDPE was presented in [6]. That study was occasioned by a practical requirement; namely, the need to replace and modernize the water network of a city with 300,000 inhabitants. Experimental measurements allowed for the mechanical identification of the materials used. The calculations were performed for two cases, for a pipe buried in the ground and for a pipe supported in a concrete massif. The advantages offered by the second solution were argued for in the work. The finite element method (FEM) was the tool used for modeling.

A frequently encountered phenomenon in the study of pipeline systems under pressure is the so-called water hammer. This phenomenon can lead to great damage, start a process of plastic deformation in the tube, or even cause the failure of the pumping system. One paper [7] proposed the use of a constructive solution that led to a reduction in the effects that the battering ram can have. This was accomplished by inserting an additional pipe. Experimental checks showed that the added polymer pipe could ensure a significant damping of the overvoltage that occurs when the mentioned phenomenon occurs. This effect must be studied because additional loads entering into the system can lead to a parrot’s beak failure. The reliability of polymer pipes made of HDPE in drinking water supply networks is an important aspect. Pipe ruptures can occur when there is a sudden overpressure or defects on the surface of the pipe. A study of this phenomenon was conducted in [8], and applied to the case of a bad water supply network. The water hammer effect that occurs in the case of transient liquid flow, although it is a rare event, can cause serious damage to a water network management system. Numerous studies have been undertaken to investigate this phenomenon. It was found that the magnitude of the event is determined by the length of the pipeline. A model that shows this was presented in [9]. The length of the pipe proved to be the decisive factor in the pressure increase time. For the different pipe lengths considered in the calculation, it was found that the differences in pressure increases can reach up to 40%, which is a significant figure that a designer must take into account. A particular case of a water supply network with HDPE pipes was studied in [10]. The work justified the use of HDPE instead of the initial solution, which was tubes made of steel. Thus, a significant reduction in project costs was achieved, and all the network conditions were fulfilled. The cost, schedule, and risk and benefit analyses justifying the use of HDPE were presented.

FEM is widely used, and allows for obtaining fast, accurate, and low-cost results for problems related to the determination of stresses and strains in a deformable body. There are a number of applications in many fields that have been solved using this method. The method can be extended to solving the problems of complex structures made up of bars, plates, or blocks.

The method is well consolidated, and the results obtained in the field have been concretized in well-known software. For the research carried out in the present work, the use of plate-type finite elements is required. In this way, information can be obtained quickly regarding the state of stresses and deformations in the structure. In FEM, a continuous structure with an infinity of unknowns (the strains or stresses at each point of the structure) is transformed into a system with a finite number of unknowns (by introducing some nodal points where the nodal displacements are known or determined). These unknowns define the degrees of freedom of the system. For each finite element, defined by its nodes, the stiffness matrix of the element is calculated, based on some approximations. Then, all the obtained matrices are assembled into a global stiffness matrix.

A simple analytical model that allows for fast and precise calculations in conditions where there is heat transfer was presented in [11]. Model verification was performed using FEM. In that work, different aspects of the problem were studied, such as the effect of the temperature of the fluid, the temperature of the soil surface, or the depth of the soil on the pipe temperature. The FEM calculation results validated the proposed model. A study of the stress and strain distribution of an HDPE pipe reinforced with polyester fibers was presented in [12] using FEM. The traction, bending, and torsional stresses were determined. The results obtained theoretically were verified experimentally. Earthquakes can cause the destruction of some pipelines due to the landslides that can occur. They can dramatically affect the life of buried pipes. In order to evaluate their effects on pipeline networks, a study is needed to study the interaction that exists between the soil and pipelines during an earthquake. This was performed by [13]. The procedures specific to this method, and an estimate of how the interaction between the soil and the pipe takes place, can provide useful results to designers. The optimization of a tube system covering underground power cables was analyzed in [14]. Pipes made of HDPE were filled with a sand–bentonite mixture to protect them from heavy mechanical loads. In the system, there was also thermal transfer through a stationary process. Using FEM, the temperature distribution in the soil, the sand–bentonite mixture, and the cables was determined. The optimal size of the cables could thus be determined. Other interesting applications of FEM in the analysis of tube systems used to transport liquids have been presented in [15,16,17,18,19,20,21,22,23,24,25,26,27,28].

The XFEM method has been used in numerous works on pipes and pipe systems, and there are many applications that have been solved with this method [29,30,31,32,33].

For HDPE, ductile failure occurs with plastic deformation, and brittle failure occurs with little or no plastic deformation. Brittle failures are thus catastrophic because they occur without warning. In this paper, a study of the emergence and development of the parrot’s beak failure, using FEM, is performed. The analysis is performed for two cases: a pipe elbow bent at 45° and a pipe segment. The results confirm that FEM is a convincing model of the way in which this failure appears and develops, and the obtained results simulate the real situation well.

## 2. Models and Methods

### 2.1. Damage of HDPE Pipes

Knowing that hydraulic loads occur when a pipeline changes direction, a problem arises in the analytical calculation of the balancing force of the hydrodynamic force.

An analytical calculation for an elbow with a deviation of α = 45° is presented in the appendix. The failure of buried pipes made of HDPE can occur for various reasons:

- Internal conditions (high pressure, rapid pressure variations, the temperature being too high or too low, and the type of liquid transported);
- Design, installation, or handling errors;
- Inappropriate material or material with initial defects;
- Geothermal activity (movement of the crust or seismic forces);
- Anthropogenic activity (heavy traffic, explosions, or other activities).

**Figure 1:**
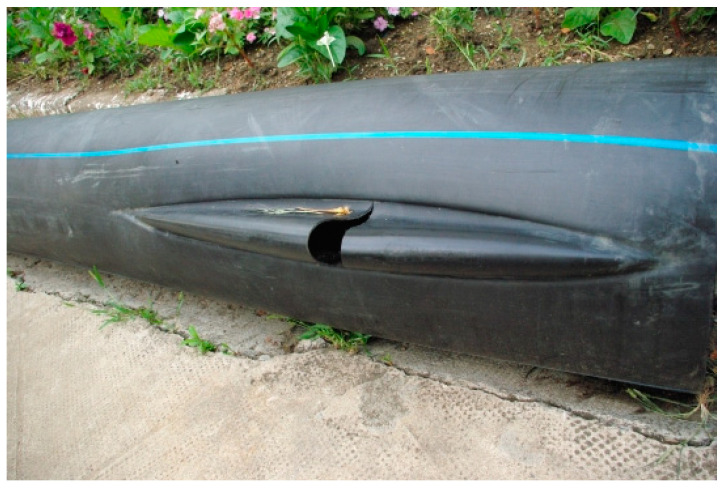
Ductile failure [3].

**Figure 2:**
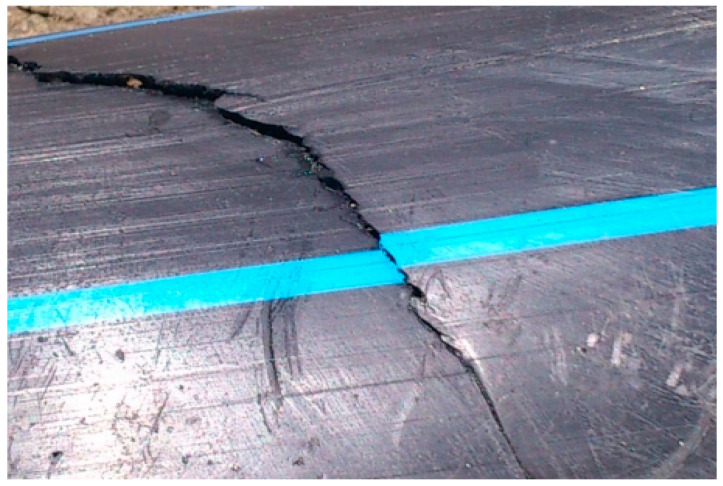
Brittle failure [4].

The common method of the deterioration of a pipe is the brittle one. A microcrack that exists after the pipe manufacturing process slowly grows in the wall. The microcracks are usually oriented along the pipe, and their opening is caused by circumferential stress (which can be caused by internal pressure or pressure from the outside). The analysis of pipes made of HDPE (presented in the introduction), established that there are three major modes of failure: ductile (mode I—Figure 1), brittle (mode II—Figure 2), and brittle/chemical (mode III).

In this work, we deal with the second mode of breaking, namely, the ductile mode. This mode is not as common as the brittle mode, but it is well known to the maintenance teams of pressure piping systems, and is called a parrot’s beak failure [34,35,36,37]. During ductile failure, the material undergoes an irreversible plastic deformation on a large scale. Usually, this defect appears in the area where the wall is thinner. There is an expansion located on a large area of the pipe. In order to avoid this type of failure, experiments are carried out on the material to determine its ductile strength over a long time. A ductile fracture must not occur before 50 years of service of the pipe. Pipe manufacturers have classified their materials, according to this requirement, into PE80 (MDPE), with a minimum admissible stress of 8.0 MPa, and PE100 (HDPE), with a minimum admissible stress of 10 MPa. Under these conditions, the lifetime must be a minimum of 50 years at 20 °C.

In this work, the occurrence of ductile failure is studied using FEM. This type of failure is important regarding the long-term service of pipes. While during the occurrence of a brittle fracture, the load can increase three times compared to the calculated value without a fracture occurring, for ductile fractures this does not happen, and the calculation must be performed with great care.

### 2.2. Experimental Methods for Determining the Mechanical Properties of the Material Used

**Figure 3:**
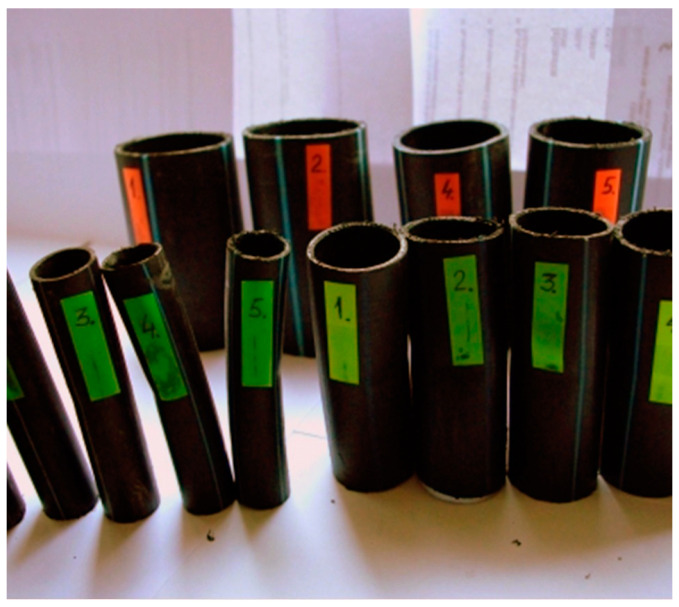
Different types of tubes used in the water supply network [4].

Due to the fact that there are no test pieces cast under pressure from the same material as that of the tubes, for the most commonly used tube sizes in the supply network, the test pieces must be cut from pipes (Figure 3).

High-density polyethylene samples were cut from the two types of tubes most often used in the distribution network:

- High-density polyethylene tube with dimensions of Φ63 × 4 mm;
- High-density polyethylene tube with dimensions of Φ90 × 6 mm.

The samples were tested under tension until breaking.

The following distributions should be known by the pipe user:

- Distribution of the longitudinal modulus of elasticity;
- Distribution of stress at the maximum load, depending on the elongation at the maximum load;
- Force distribution at the maximum load, depending on the deformation at the maximum load;
- Distribution of the longitudinal modulus of elasticity, depending on tensile strength;
- Distribution of stiffness, depending on the force at the maximum load;
- Distribution of stress at maximum deformation, according to the elongation at maximum deformation;
- Distribution of the breaking force, depending on the breaking deformation;
- Distribution of the breaking stress, according to the breaking elongation.

These distributions are shown graphically in Figure 4, Figure 5, Figure 6, Figure 7, Figure 8, Figure 9, Figure 10 and Figure 11. The manufacturer should also make them known to those who are going to use the tubes in a network.

Based on laboratory measurements, the following conclusions can be drawn:

- As the tube diameter increases, the standard deviation of the values increases;
- The values of Young’s modulus fell between 1500 and 4000 MPa for the high-density polyethylene samples taken from the 90 × 6 mm tube, and between 1000 and 1400 MPa for the high-density polyethylene samples taken from the 63 × 4 mm tube;
- The average stress at the maximum load, depending on the elongation at the maximum load, was around 20 MPa for both types of samples;
- The force at the maximum load for the high-density polyethylene cut from the 90 × 6 mm tube was between 1.2 kN and 1.6 kN, and for the case of the high-density polyethylene cut from the 63 × 4 mm tube, it was between 0.7 kN and 0.8 kN;
- The most important scattering of values was obtained for the distribution of the stress at the maximum deformation, according to the displacement at the maximum deformation for both types of specimens made of high-density polyethylene tubes.

## 3. Results

### 3.1. The Case of Elbow DN 315 Buried in Soil

**Figure 12:**
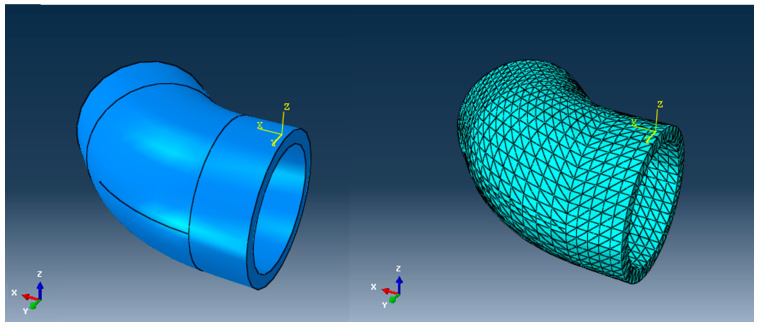
The geometric model of the unsupported and the discretized analysis model of the DN 315 elbow.

In what follows, we analyze, via the FEM, two cases of the appearance of a parrot’s beak-type break, one for an elbow and another for a linear tube, subjected to the usual pressures found in water transport networks. The mode of the appearance and propagation of the crack are studied. The geometric model of the DN 315 elbow made of high-density polyethylene that is unsupported in a concrete anchor mass is shown in Figure 12. The model will be used to simulate the failure of the elbow. The discretized analysis model is presented in the same figure.

The discretization of the structural model was performed considering a total number of 14,985 nodes, and a total number of 12,370 elements, of which 12,174 were parallelepiped elements of the C3D8R type, and 196 were prismatic elements of the C3D6 type. The degrees of freedom (DOFs) were the displacements in the nodes (three translations). For greater accuracy in the representation of the stresses in the bend, in a second version two elements per thickness were used. The influences of the weight of the concrete and the elbow and the water column were not considered.

The model required for the analysis has, as the input data, the pressure inside the tube, taken as the reference pressure perpendicular to the inner wall of the tube; the fittings; and the tube ends—so that all the degrees of freedom are cancelled out. Abaqus/CAE 6.14-5 with XFEM is used in the numerical analysis of the crack. The simulation is performed on DN 315 arteries. The tube model is created using an extruded solid body. The chosen material of the tube is HDPE. The steps followed in the analysis are as follows: create the solid; create the strip crack; create the section and set material properties; create an additional crack scheduling step; create a crack interaction; create a unitary assembly from the solid and crack strip; create embeds; create a load on the inner surface; discretize the model; choose output calculation parameters; run the simulation; and obtain results.

In the analysis of the tubes, only the stresses and deformations caused by the internal pressure to which they are subjected in a water distribution network are monitored.

**Figure 13:**
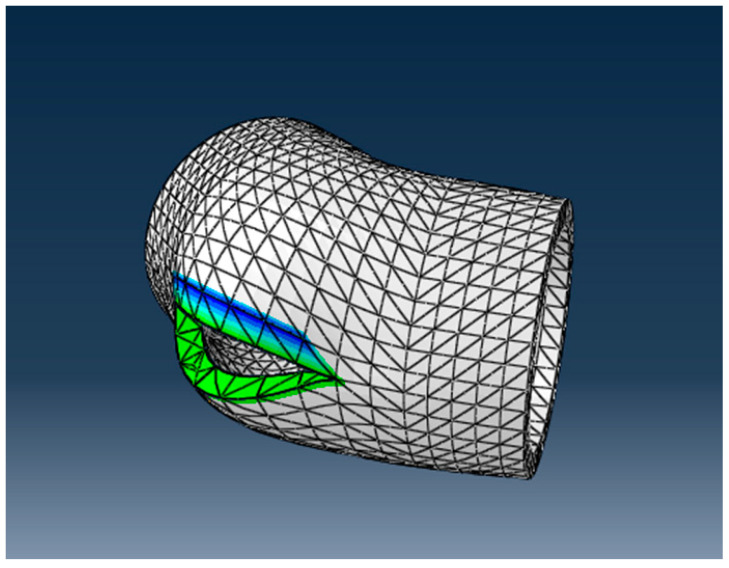
Analysis model of DN 315 elbow crack.

**Figure 14:**
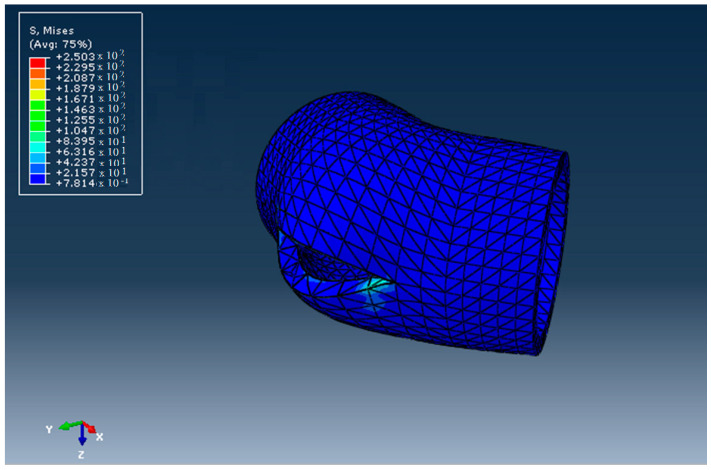
The stress field in the DN 315 elbow.

**Figure 15:**
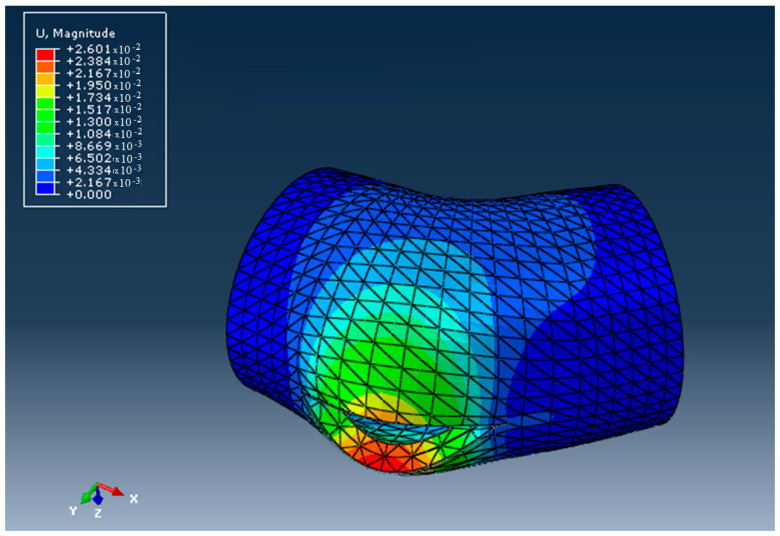
The displacement field in the DN 315 elbow.

The aspect of the elbow after the ductile fracture, obtained using Abaqus/CAE 6.15-XFEM, is presented in Figure 13. The stress distribution is shown in Figure 14. The stress at which the break occurred was 517 MPa > 21.9 Mpa, which is larger than the admissible stress for the HDPE materials used. The deformations of the elements that discretized the elbow around the crack are shown in Figure 15.

### 3.2. FEM Analysis of the Pressurized HDPE-DN 315 Tube

The tube under analysis has the following as input data:

- The pressure inside the tube, taken as the reference pressure perpendicular to the inner wall of the tube;
- The fixed ends of the tube and the forces that act on the tube, so that equilibrium is assured.

**Figure 16:**
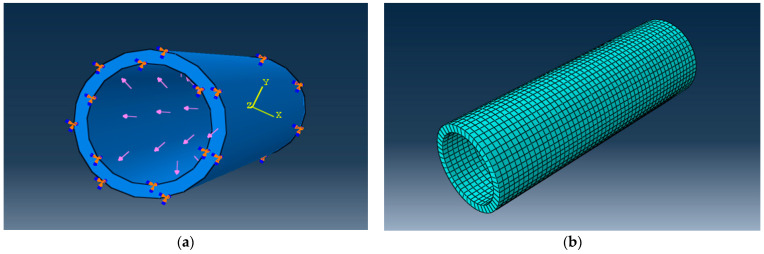
Geometrical (**a**) and FEM model (**b**) of the DN 315 tube subjected to internal pressure.

The dimensions and material constants are as follows: the outer diameter, D_out_ = 315 mm; the inner diameter, D_in_ = 257.00 mm; the wall thickness, Thk = 28.60 mm; the density of the D_tube_ material = 970 kg/m^3^; Young’s modulus of the E_tube_ = 1300 MPa; Poisson’s ratio ν of the tube = 0.33. The internal pressure in the tube produces radial and circumferential stresses, as shown in Figure 16.

To perform the numerical analysis with FEM using ABAQUS 2020, the discretized model was presented (see Figure 16b). The discretization was performed using C3D8R-type parallelepiped elements; 2300 elements were generated for the obtained model.

**Figure 17:**
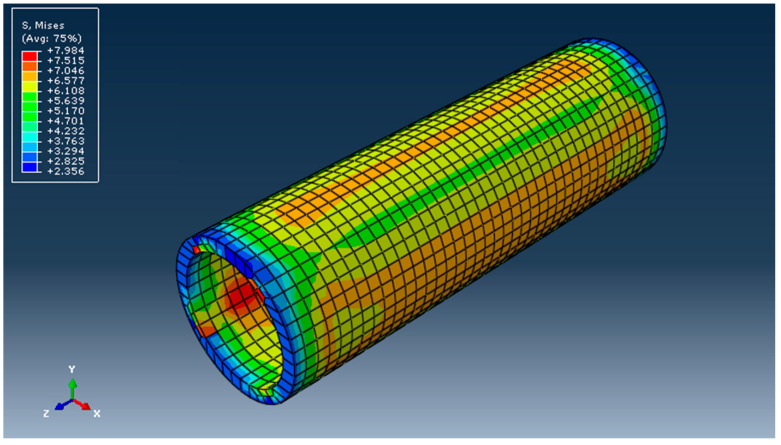
Stress field in tube DN 315 in MPa (von Mises).

Figure 17 shows the stress distribution obtained after performing the calculation.

**Figure 18:**
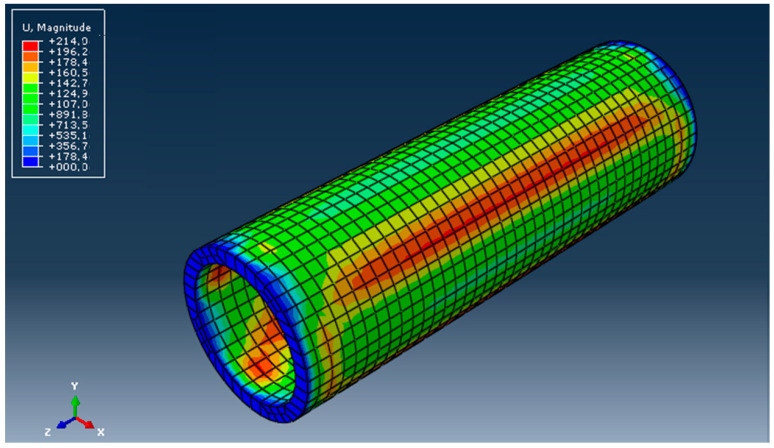
The displacement field in mm.

The field of displacements is presented in Figure 18.

Abaqus/CAE 6.14-5 with XFEM was used in the numerical analysis of the crack. The steps followed in the analysis are as follows: solid generation; the creation of a crack strip; the creation of section and setting material properties; the creation of an additional step for crack programming; the creation of crack-type interaction; the creation of unitary assembly from the solid and crack strip; the creation of embeds; the creation of a load on the inner surface; the discretization of the model; the choice of output calculation parameters; the execution of the simulation; and the obtaining of results.

**Figure 19:**
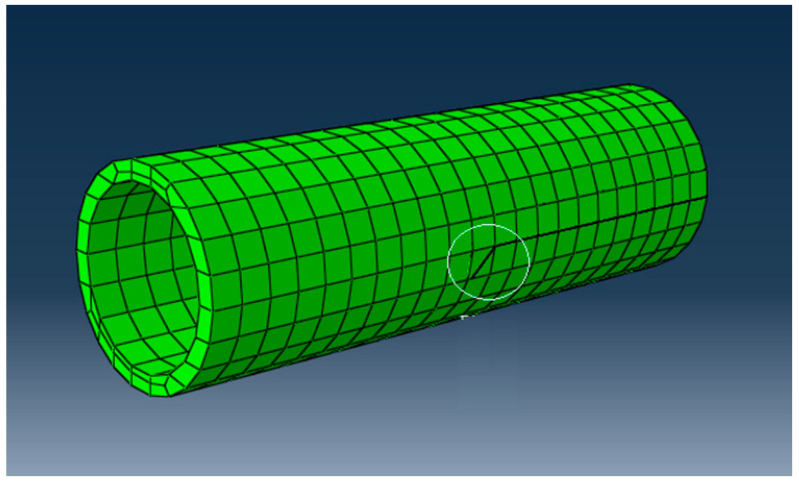
Tube-crack-analysis model, DN 315.

In the analysis of the tubes, only the stresses and deformations caused by the internal pressure to which they are subjected in a water distribution network were monitored. The model that created the crack-type interaction, as well as the crack strip, is presented in Figure 19.

**Figure 20:**
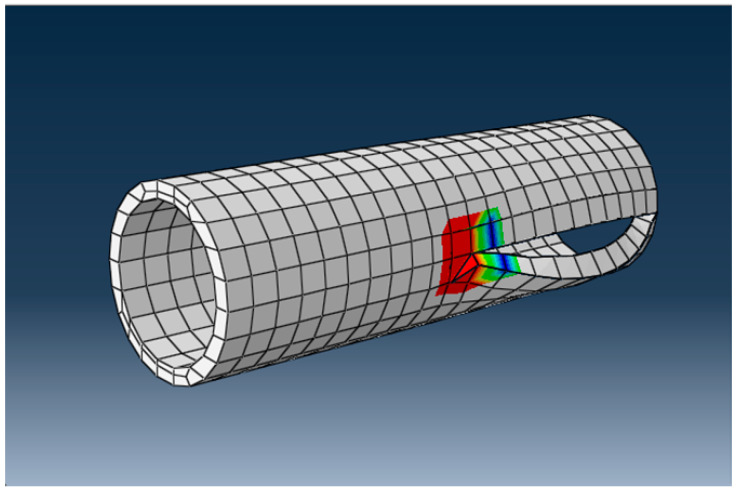
The appearance of the crack after breaking in the DN 315 tube.

The break is ductile and resembles the “parrot’s beak” presented previously. After the calculation, it was found that the stress at which the break occurred was 8563 MPa, a much higher value than the admissible stress at break for HDPE. The appearance of the crack after breaking is shown in Figure 20.

**Figure 21:**
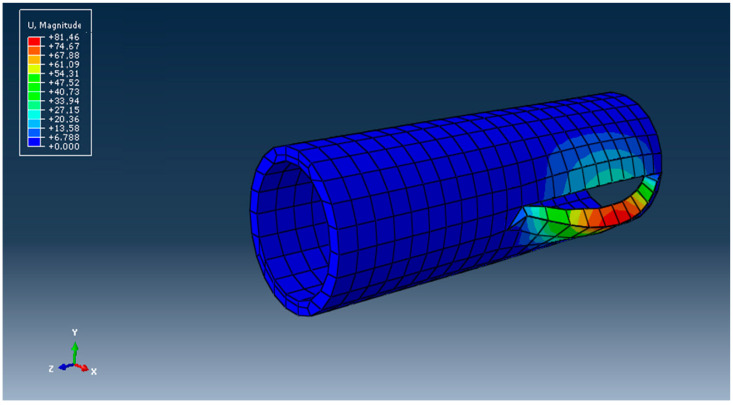
Displacements and growth of the crack in the wall of the DN 315 tube.

The displacements of the finite-element nodes near the crack are shown in Figure 21.

Following the analysis, it can be seen that FEM is a very useful tool for the study of this type of failure, and it is possible to follow the initiation and the growth of cracks in tube walls.

## 4. Discussion and Conclusions

The main objective of this work was to study the development of failure in HDPE tubes for the case of the formation of the so-called parrot’s beak.

Analyzing the results obtained after the calculations using FEM for the two types of pipes reveales that this method can well describe the origin and evolution of a crack that appears as a result of a parrot’s beak failure. Pipes made of HDPE present significant advantages compared to other common materials which are currently used: long service life; cathodic protection; no anticorrosion protection is necessary; low maintenance costs; good hydraulic properties; easy design, manufacture, and installation; proper sealing at high pressures; low weight; low logistical costs; and the possibility of making long tube lengths (and, therefore, fewer connections). It is obvious that, over time, HDPE pipe networks have come to have impressive resistance properties, ensuring their long-term service. Accidents and breakages can occur for various reasons, even under these conditions: design and manufacturing mistakes, mistakes made in the installation of the system, incorrectly chosen materials, thermal exposure, shocks during operation, chemical exposure, extreme natural phenomena, soil quality, etc. Malfunctions can lead to explosions, fires, loss of life, and property damage, so it is very important to address them. As a result, failure analysis must represent a standard practice in the field.

The parrot’s beak failure represents a second cause of the destruction of water supply networks; therefore, the establishment of powerful calculation methods is an objective to which the authors have tried to contribute in the present work.

## Figures and Tables

**Figure 4:**
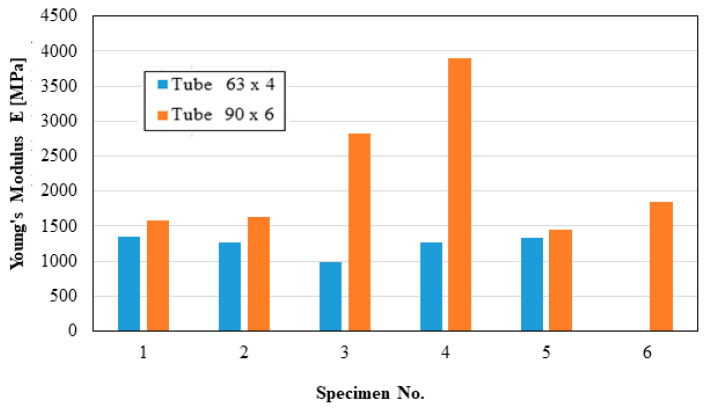
Young’s modulus.

**Figure 5:**
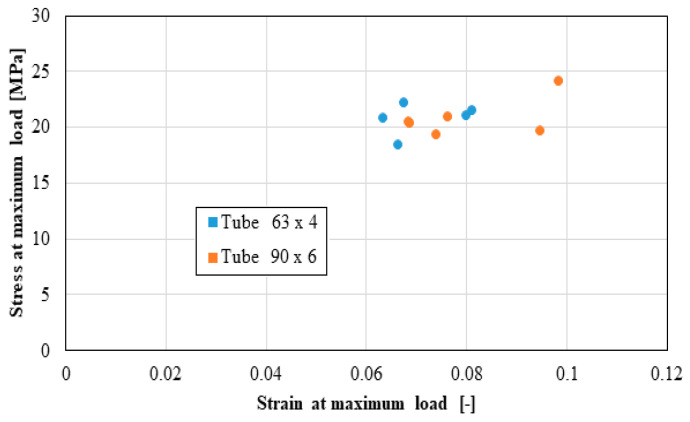
Stress at maximum load.

**Figure 6:**
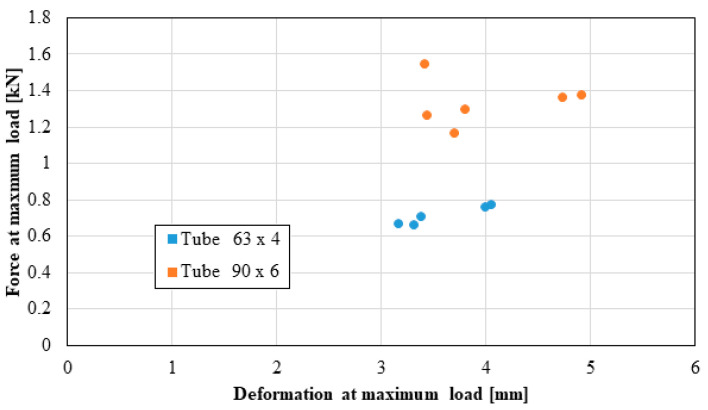
Force at maximum load versus deformation at maximum load.

**Figure 7:**
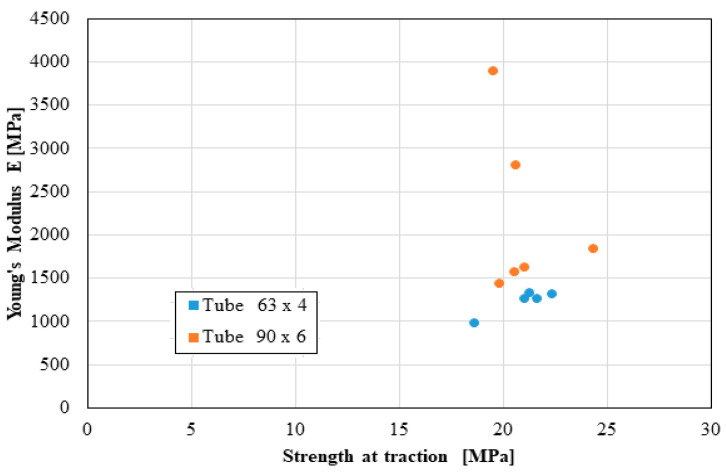
Young’s modulus versus strength at traction.

**Figure 8:**
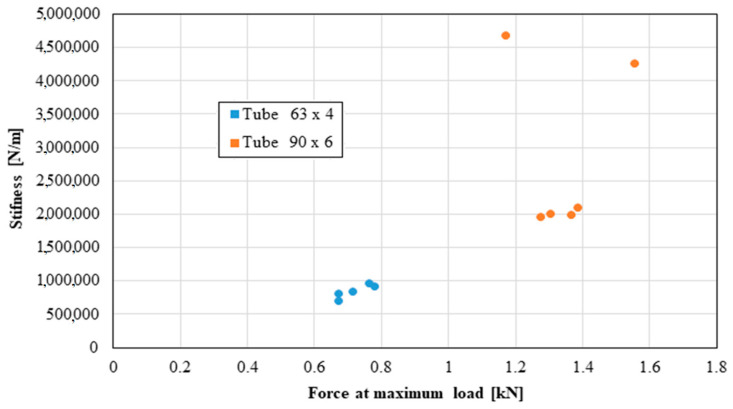
Stiffness versus force at maximum load.

**Figure 9:**
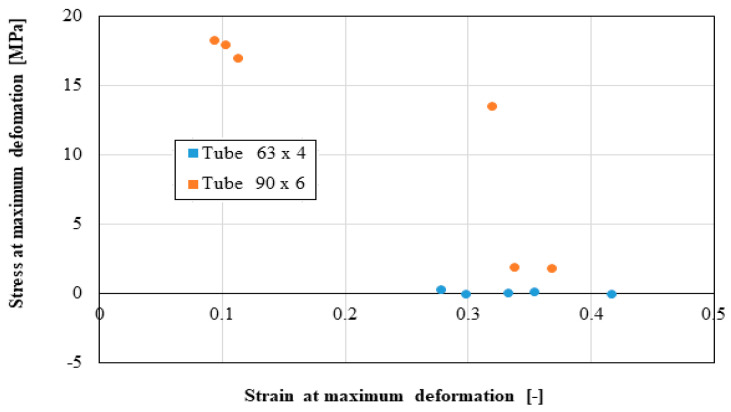
Stress at maximum deformation versus strain at maximum deformation.

**Figure 10:**
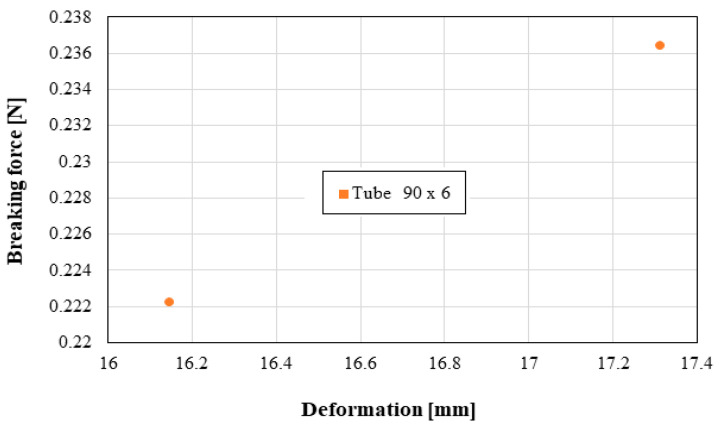
Breaking force versus deformation.

**Figure 11:**
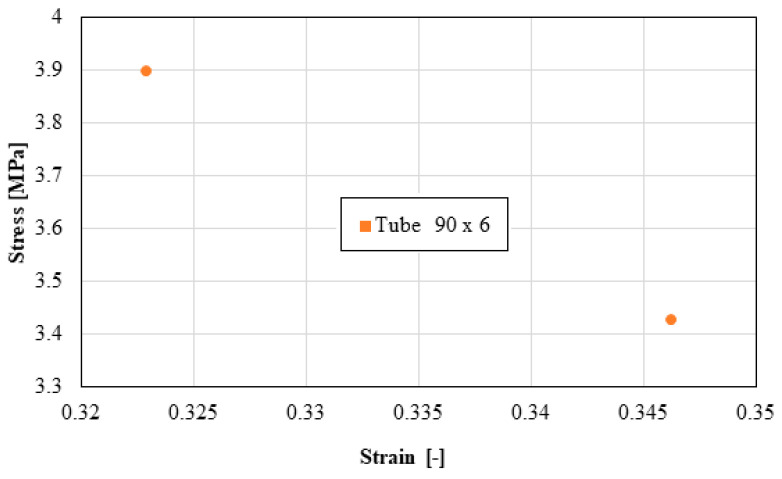
Stress versus strain.

## Data Availability

Not applicable.
